# Bridging Autoimmunity and Hyperinflammation: Shared Immunological Pathways Among Autoimmune/Inflammatory Syndrome Induced by Adjuvants, Systemic Lupus Erythematosus, and Macrophage Activation Syndrome

**DOI:** 10.3390/biomedicines14092101

**Published:** 2026-09-17

**Authors:** Gabriela D’El Rei Tamaki, Guilherme de Oliveira Vitiello, Érika Bevilaqua Rangel

**Affiliations:** 1 Paulista School of Medicine, Federal University of São Paulo, São Paulo 04038-031, SP, Brazil; 2 Department of Medicine, Nephrology Division, Federal University of São Paulo, São Paulo 04038-031, SP, Brazil; 3 Instituto Israelita de Ensino e Pesquisa Albert Einstein, Hospital Israelita Albert Einstein, São Paulo 05652-900, SP, Brazil

**Keywords:** autoimmune/inflammatory syndrome induced by adjuvants (ASIA), systemic lupus erythematosus (SLE), macrophage activation syndrome (MAS), cytokines, interleukins

## Abstract

Autoimmune/inflammatory syndrome induced by adjuvants (ASIA) is characterized by immune dysregulation associated with exposure to exogenous substances with immunostimulatory properties, particularly in susceptible individuals. Systemic lupus erythematosus (SLE) is a complex and heterogeneous autoimmune disease characterized by loss of immune tolerance, autoantibody production, and immune complex deposition. Macrophage activation syndrome (MAS) is a life-threatening hyperinflammatory syndrome characterized by excessive activation of macrophages and T lymphocytes and may occur as a severe complication of SLE and other inflammatory conditions. This review explores the clinical and immunological overlap among ASIA, SLE, and MAS, with particular attention to the potential contribution of adjuvant-related exposures to immune activation in susceptible individuals. Shared and convergent immunological pathways, including activation of innate and adaptive immunity, loss of immune tolerance, macrophage activation, and dysregulation of cytokine networks involving IL-1β, IL-6, IL-17, IL-18, TNF-α, and interferon-γ, are discussed. Rather than representing an established causal or sequential pathway, the relationship among these conditions is considered within a conceptual framework based on overlapping immunological mechanisms. Clinically, overlapping manifestations among ASIA, SLE, and MAS may pose diagnostic challenges and contribute to underrecognition or delayed treatment. Careful assessment of potential triggering exposures, integration of clinical and laboratory findings, and prompt recognition of hyperinflammatory manifestations are important for appropriate clinical management. This review highlights the need for further mechanistic and prospective studies to determine the clinical relevance of the immunological overlap among ASIA, SLE, and MAS, validate potential biomarkers, and evaluate targeted therapeutic strategies directed at shared inflammatory pathways.

## 1. Autoimmune/Inflammatory Syndrome Induced by Adjuvants (ASIA)

### 1.1. Definition

First described by Shoenfeld and Agmon-Levin in 2011, Autoimmune/Inflammatory Syndrome Induced by Adjuvants (ASIA) refers to a group of immune-mediated conditions that arise in susceptible individuals following exposure to an adjuvant, that is, substances or materials that enhance immune responses to an antigen, primarily through activation of innate immunity [1]. These immunogenic substances, including silicone implants, aluminum salts, and mineral oil, among others, have been associated with an increased risk of immune hyperactivation, leading to autoantibody production and the development of autoimmune disorders in predisposed individuals [2].

More than 4400 documented cases have been reported since ASIA’s recognition in 2011 [3]. The diagnosis of ASIA requires fulfillment of either two major criteria or one major plus two minor criteria. Major criteria include [2]: (i) exposure to an external stimulus prior to the onset of clinical manifestations; (ii) development of “typical” clinical manifestations such as chronic fatigue with nonrestorative sleep or sleep disturbances, myalgia, myositis or muscle weakness, arthralgia and/or arthritis, cognitive impairment and memory loss, pyrexia, sicca symptoms (dry mouth and dry eyes), and neurological manifestations, particularly when associated with demyelination; (iii) clinical improvement after removal of the triggering agent; and (iv) typical biopsy findings in affected organs. Minor criteria include: (i) the appearance of autoantibodies or antibodies directed against the suspected adjuvant; (ii) other clinical manifestations (e.g., irritable bowel syndrome or Raynaud phenomenon); (iii) specific HLA (Human Leukocyte Antigen) associations (such as HLA-DRB1 and HLA-DQB1); and (iv) progression to a defined autoimmune disease, such as multiple sclerosis, rheumatoid arthritis, Sjögren’s syndrome, or systemic sclerosis.

### 1.2. Epidemiology and Triggers of ASIA

Although large epidemiological studies assessing the prevalence and incidence of ASIA are lacking, several reports have addressed this issue [4,5,6,7]. A wide range of adjuvants has been associated with ASIA, with vaccines (e.g., HPV, HBV, influenza) and silicone implants being the most extensively studied. Other materials include mineral oil, hyaluronic acid, polyalkylimide, polyacrylamide gel, and collagen.

The mean age at ASIA diagnosis was 37.6 years (range, 4 to 82 years old) [5]. There is a marked female predominance, accounting for up to 86.7% of cases in published series. Notably, 89% of patients diagnosed with ASIA subsequently develop a defined rheumatic/autoimmune disease, with a mean interval of 16.8 months (ranging from 3 days to 5 years) between adjuvant exposure and autoimmune disease onset [5]. These conditions span a broad spectrum, including vasculitis (such as Behçet disease and giant-cell arteritis), rheumatologic disorders (such as Sjögren’s syndrome, undifferentiated connective tissue disease, systemic lupus erythematosus [SLE], antiphospholipid syndrome, systemic sclerosis, and rheumatoid arthritis), endocrine diseases (such as Hashimoto thyroiditis and type 1 diabetes mellitus), and sarcoidosis, among others [3,5,8,9].

Multiple vaccines have also been associated with reported manifestations of ASIA, with an increased number of cases described following COVID-19 vaccination. Although the immunological mechanisms underlying vaccine- and adjuvant-induced immune responses remain incompletely understood, and epidemiological studies have not demonstrated a statistically significant association with most post-vaccination adverse events, a causal relationship cannot be entirely excluded in susceptible individuals. This has been proposed for selected conditions, such as somatoform neuropsychiatric syndrome following HPV-16/18 vaccination [10].

In the context of COVID-19 vaccination, both inactivated and mRNA vaccines have been associated with ASIA manifestations, including subacute thyroiditis occurring 4–7 days after vaccination [11]. The postpartum period may represent a predisposing factor for the development of ASIA following vaccination [11].

Among the reported adjuvants, mineral oil has been particularly implicated in cases of mineral oil-associated ASIA (ASIA-MO), owing to its ability to act as a proinflammatory foreign body, promoting continuous release of proinflammatory cytokines and phagocytic dysfunction. Accordingly, individuals chronically exposed to mineral oil may fulfill the criteria for autoimmune diseases, including SLE, rheumatoid arthritis, and systemic sclerosis, among others [12]. ASIA-MO occurrence appears to be particularly concentrated in Latin America [2]. The clinical presentation is heterogeneous and varies in complexity and severity, similar to ASIA triggered by other adjuvants. Notably, up to 20.8% of cases of ASIA have been linked to mineral oil exposure [4], and approximately 40% of patients with ASIA-MO develop a defined autoimmune disease, such as SLE, systemic sclerosis, or rheumatoid arthritis [12].

### 1.3. Pathophysiology of ASIA

ASIA is characterized by chronic activation of both innate and adaptive immunity, leading to autoantibody production, persistent inflammation, and systemic manifestations in individuals with genetic predisposition (particularly HLA-DRB1 and PTPN22) [3,13]. This process is sustained by prolonged antigenic stimulation, which prevents restoration of immunologic homeostasis and promotes either de novo autoimmunity or reactivation of preexisting autoimmune diseases [2]. However, the pathophysiology of ASIA remains incompletely understood, largely due to the diversity of adjuvants and clinical phenotypes involved. However, available evidence suggests that ASIA represents an immune amplification process initiated by adjuvant stimulation, through exogenous material or persistent exposure, leading to activation of innate immunity and generation of a proinflammatory microenvironment. This process recruits neutrophils, monocytes/macrophages, and particularly dendritic cells. These antigen-presenting cells capture antigens, undergo maturation, and upregulate costimulatory molecules, releasing cytokines that shape the adaptive immune response: interleukin (IL)-1β and IL-18 (associated with cytosolic inflammatory platforms), tumor necrosis factor-alpha (TNF-α), and IL-6 (sustaining inflammation and lymphocyte activation), as well as polarization pathways mediated by IL-12 (Th1 skewing with [interferon-gamma] IFN-γ production and macrophage/cytotoxic T lymphocyte CD8^+^ activation), IL-4/IL-5/IL-13 (Th2 skewing with B-cell activation and antibody production), and IL-23 plus transforming growth factor-β [TGF-β] (favoring Th17 differentiation, IL-17 production, neutrophil recruitment, and tissue inflammation). In susceptible individuals, this environment may lead to regulatory T-cell dysfunction, polyclonal B-cell activation, and enhanced autoantigen presentation, facilitating bystander activation and epitope spreading, thereby increasing the likelihood of autoantibody production and progression to a defined autoimmune phenotype [9,14].

## 2. The Interface Between Autoimmune/Inflammatory Syndrome Induced by Adjuvants (ASIA) and Systemic Lupus Erythematosus (SLE)

SLE affects more than 3.4 million individuals worldwide [15] and occurs predominantly in women, at a ratio of approximately 9:1 compared with men, and in Black individuals at roughly twice the rate observed in White populations [16,17]. Although less frequent in men, SLE in males tends to be more severe and is more strongly associated with adverse outcomes, including lupus nephritis [18]. Clinical manifestations typically begin in young individuals, most commonly between 15 and 44 years of age [19]. The clinical presentation is highly heterogeneous and may include constitutional, hematologic, neuropsychiatric, mucocutaneous, musculoskeletal, renal, and serosal manifestations. According to the 2019 European League Against Rheumatism (EULAR)/American College of Rheumatology (ACR) classification criteria, SLE is classified with a minimum score of 10 points, provided that at least one clinical criterion is present and antinuclear antibodies (ANA) are positive at a titer ≥ 1:80 [20].

SLE is a systemic autoimmune disorder characterized by the production of pathogenic autoantibodies (such as anti-double-stranded DNA and anti-Sm antibodies) and the formation of circulating immune complexes that deposit in tissues, leading to inflammation and organ damage [21]. Its clinical spectrum is broad, encompassing cutaneous, hematologic, articular, neurologic, and renal manifestations, with lupus nephritis representing a major cause of morbidity and mortality. The interplay among genetic predisposition, environmental triggers, and acquired immune dysfunction is central to its pathogenesis, and adjuvant agents such as mineral oil may act as potent triggers of this immune dysregulation [2].

In SLE, the loss of immune tolerance results in autoantibody production, particularly against nuclear antigens, formation of circulating immune complexes, and their deposition in tissues, leading to complement activation and chronic inflammation. Briefly, the loss of immune tolerance in SLE can be summarized in three main mechanisms: (i) efferocytosis defect (impaired clearance of apoptotic cells by macrophages and phagocytes); (ii) apoptosis defect, primarily affecting B-cell proliferation and tolerance (both central and peripheral); and (iii) inappropriate activation of type I interferon responses (“type I interferonopathy”), characterized by IFN-α production by plasmacytoid dendritic cells, interferon gene signatures, and nucleic acid sensing pathways, including TLR7/TLR9 and cGAS-STING, as previously reviewed [22,23,24,25,26].

This immunopathologic cascade recurrently affects the skin, joints, kidneys, and other organs, with a heterogeneous clinical course characterized by flares and remissions. Persistent or poorly controlled inflammatory activity may result in cumulative organ damage and, in severe cases, major complications and increased early mortality [17].

Moreover, SLE arises in a multifactorial context involving genetic and epigenetic components, as well as environmental triggers that promote autoimmunity. The accumulation of autoantigens derived from impaired clearance of apoptotic cells, such as may occur with altered macrophage function, and their presentation to the immune system in predisposed individuals lead to the formation of pathogenic autoantibodies. Patients with SLE exhibit reduced IL-2 production by CD4^+^ T lymphocytes, weakening regulatory T-cell (Treg) function and permitting inflammatory effector responses. Concurrently, IL-17 levels are increased, contributing to macrophage recruitment and tissue inflammation [27]. Together with elevated IL-21, IL-17 promotes plasma cell differentiation and thereby enhances pathogenic autoantibody production [28]. This Th17/IL-17 axis is sustained by IL-23 [29]. At the same time, serum IL-10 levels are consistently elevated and correlate with disease activity, potentially stimulating polyclonal B-cell populations and promoting autoantibody production [30]. At the apex of the innate–adaptive immune interface lies the type I interferon signature: IFN-α levels are elevated in active lupus, and interferon-regulated genes are upregulated in peripheral blood mononuclear cells [31,32,33]. In target organs, proinflammatory cytokines amplify tissue injury; cytokines such as IL-6, IL-17, type I interferons, and TNF-α contribute to immune dysfunction, local inflammation, and organ damage, in addition to immune complex deposition. In the kidney, resident cells may respond to and amplify injury, for example, mesangial cells produce IL-6 [34].

Specifically regarding the development of SLE, the association with mineral oil appears to be well established in the literature, and the pathophysiological correlation may reside in the previously described immunologic cascade involving IL-1β, IL-18, IL-17, IL-23, IL-4, IL-5, IL-6, IL-13, TNF-α, IFN-γ, and TGF-β [5,6]. However, additional mechanistic associations have been described in earlier studies: IL-6 has been identified as essential for anti-chromatin/anti-dsDNA production, whereas interferons are critical for anti-nRNP/Sm and anti-Su antibodies. Some experimental models demonstrate IL-4 and IL-6 accompanying spontaneous autoantibody production, but hydrocarbon exposure may shift the immune balance toward IFN-γ and IL-12, thereby altering the autoantibody repertoire. Moreover, certain oils induce delayed IL-6 and TNF-α production, suggesting sustained inflammation that favors the anti-DNA/chromatin axis [35,36,37,38,39].

In fact, studies from the late 20th and early 21st centuries had already demonstrated in animal models that mineral oil administration was associated with the production of antinuclear autoantibodies (e.g., anti-dsDNA and anti-chromatin antibodies), which are intrinsically linked to SLE [35,36,37,38,39]. Subsequently, mineral oil was associated with ASIA and with the development of autoimmune diseases in both animal models and humans [12,40,41,42,43]. In line with these findings, SLE has been reported in approximately 3–3.6% of ASIA cases [4,6].

The immunopathological link between ASIA and SLE may also involve basophils, which have emerged as key amplifiers of autoimmune responses after immune tolerance has been disrupted. During active SLE, basophils are activated by autoreactive IgE antibodies directed against nuclear antigens, particularly anti-dsDNA IgE. Upon activation, basophils promote B-cell survival and differentiation through IL-4 secretion, membrane-bound BAFF (B-cell activating factor), and IgE-dependent signaling, thereby enhancing plasma cell differentiation and autoantibody production [44]. Simultaneously, prostaglandin D2 (PGD2) induces the upregulation of homing receptors, including CD62L, CCR7, and CXCR4, promoting basophil migration from the peripheral blood to secondary lymphoid organs [45]. Within these tissues, basophils further amplify humoral immunity by secreting IL-4 and IL-6, expressing BAFF, and promoting T follicular helper (TFH2) cell differentiation through IL-4 and PD-L1, ultimately driving the expansion of autoreactive B cells [46]. In parallel, basophils also favor Th17 differentiation and IL-17 production, further exacerbating systemic inflammation and tissue injury [47]. The resulting autoantibodies form immune complexes that deposit within the renal glomeruli, initiating the inflammatory cascade characteristic of lupus nephritis [48].

Basophil migration to lymphoid tissues results in peripheral basopenia, a finding consistently associated with disease activity in patients with SLE and independent of immunosuppressive treatment, reflecting cellular redistribution rather than depletion [49].

Experimental evidence further supports the pathogenic role of basophils, as both spontaneous lupus-prone (Lyn-deficient) and pristane-induced murine models demonstrate that basophil depletion prolongs survival, improves renal function, and reduces circulating autoantibodies and IL-17 levels, whereas adoptive transfer of basophils accelerates disease progression [46,48].

Collectively, these findings suggest that peripheral basophil counts may serve as a surrogate biomarker of SLE activity, including lupus nephritis, and identify the PGD2–basophil signaling axis as a promising therapeutic target warranting further investigation.

To further explore the potential association among SLE and ASIA, we conducted a focused literature search in PubMed using combinations of the terms “SLE” and “ASIA”. In addition, Open Evidence was consulted using the query “SLE after adjuvant exposure” to identify potentially relevant clinical evidence. The available literature indicates that reports directly linking adjuvant exposure and ASIA to subsequent SLE remain limited and consist predominantly of observational studies, case series, and individual case reports.

In Table 1 [50,51], we summarize the main findings on bioimplant-induced ASIA and SLE, including clinical and laboratory characteristics, as well as treatment outcomes.

Notably, the case reported by Vitiello et al. represents, to our knowledge, the only clinical report describing the concomitant occurrence of ASIA, SLE, and MAS. Therefore, although this case provides clinical support for the biological mechanism of an overlap among these conditions, it should not be interpreted as evidence establishing a causal or sequential ASIA–SLE–MAS pathway. Rather, it should be considered hypothesis-generating evidence supporting the broader mechanistic framework proposed in this review, which is based on shared pathways involving innate and adaptive immune activation, loss of immune tolerance, macrophage activation, and cytokine dysregulation.

Additionally, ASIA and SLE can be triggered not only by bioimplants but also by various vaccines, as summarized in Table 2 [53,54,55,56,57].

Specifically, pharmacovigilance analyses, such as the VAERS-based study by Xie et al., are valuable for detecting potential safety signals in very large populations but rely on spontaneous reporting and are susceptible to underreporting, reporting bias, incomplete clinical information, and the absence of an appropriate unvaccinated comparator [53]. Consequently, disproportionality signals indicate reporting associations and should not be interpreted as demonstrating causality. In contrast, case–control studies include comparator groups and allow adjustment for potential confounders, providing more robust estimates of relative risk, although they remain susceptible to selection, recall, and residual confounding. Meta-analyses provide greater statistical power by pooling evidence across studies; however, their conclusions depend on the quality, design, populations, vaccines, exposure windows, and outcome definitions of the included studies, and substantial between-study heterogeneity may affect pooled estimates.

Importantly, the large pharmacovigilance analysis included in Table 2 identified 1976 lupus reports among 11,301,263 vaccine-related adverse events, corresponding to an absolute reported frequency of only 0.017% [53]. This very low frequency should be appropriately contextualized and should not discourage vaccination, given the well-established benefits of vaccines in preventing serious infectious diseases. Furthermore, the evidence is not consistent across study designs: the 2024 meta-analysis found no significant overall association between vaccination and SLE (OR 1.14, 95% CI 0.86–1.52) [54], whereas the international case–control study reported an adjusted OR of 0.9 (95% CI 0.5–1.5) [56]. Therefore, epidemiological studies summarized in our review have not demonstrated a consistent increase in SLE risk following vaccination.

Rather than routine additional surveillance of the general population, heightened clinical awareness may be appropriate in individuals with a history of autoimmune disease, previous immune-mediated reactions to vaccines or adjuvants, or other potential susceptibility factors. In such individuals, new persistent systemic manifestations following vaccination should prompt appropriate clinical evaluation, while temporal association alone should not be considered evidence of causation. Importantly, the incidence of vaccine-associated ASIA–SLE–MAS as a combined phenotype remains unknown because clinical evidence directly linking all three conditions is currently insufficient.

Prospective pharmacovigilance studies incorporating standardized diagnostic criteria, appropriate comparator populations, and molecular or immunological biomarkers are needed to characterize susceptible populations and determine the true incidence and clinical significance of these exceptionally rare events.

## 3. The Interface Among Autoimmune/Inflammatory Syndrome Induced by Adjuvants (ASIA), Systemic Lupus Erythematosus (SLE), and Macrophage Activation Syndrome (MAS)

In severe cases of active autoimmunity, the inflammatory response may become hyperinflammatory, culminating in macrophage activation syndrome (MAS), a life-threatening condition characterized by uncontrolled activation of cytotoxic cells, including T lymphocytes and natural killer (NK) cells, as well as myeloid cells such as macrophages and dendritic cells of macrophages, massive cytokine release, and multiorgan failure [58].

MAS is characterized by dysregulated immune activation, leading to histiocytic activation and hemophagocytosis (phagocytosis of erythrocytes, lymphocytes, or other precursors by activated macrophages in bone marrow as a result of cytokine storm). This results in cytopenias, persistent fever, hepatosplenomegaly, and organ dysfunction [59]. MAS represents a specific form of secondary hemophagocytic lymphohistiocytosis (HLH), frequently associated with rheumatologic, infectious, or neoplastic diseases, and characterized by less prominent genetic contribution compared with primary HLH [60].

Despite advances in the understanding of MAS pathophysiology, diagnosis remains challenging, largely due to the lack of disease-specific diagnostic criteria for MAS in the context of rheumatic diseases, which often hampers early recognition [58]. However, useful tools can support diagnostic confirmation, including the HScore, which estimates the probability of MAS/secondary HLH based on nine variables [61], and the HLH-2004 criteria, which require at least five of eight features [59]: fever, splenomegaly, cytopenias affecting at least two of three peripheral blood lineages, hypertriglyceridemia and/or hypofibrinogenemia, hemophagocytosis in bone marrow, spleen, or lymph nodes, low or absent NK-cell activity, hyperferritinemia, and elevated soluble IL-2 receptor (sIL-2R).

Although MAS is frequently associated with other rheumatologic conditions, the occurrence of MAS in SLE ranges from 0.9% to 4.6% [17], with mortality approaching one-third of patients [62], whereas the proportion of SLE among MAS cases is close to 50% [63]. Nevertheless, the true incidence may be underestimated because MAS can be misclassified as lupus flares or complications of the underlying disease [62], particularly given that both entities share overlapping cytokine signatures and clinical manifestations [63].

In patients with SLE-associated MAS, 54.65% presented with MAS as the initial manifestation of SLE, while 45.35% developed MAS during the course of SLE, indicating that this complication can arise at any stage of disease [64]. In contrast, a population-based study including more than 200,000 individuals between 2001 and 2013 found no statistically significant association between prior MAS and subsequent development of SLE but demonstrated that the risk of MAS was substantially higher (up to sevenfold) in patients with established SLE; notably, 1.19% of SLE patients developed MAS [63].

Moreover, SLE activity was increased in all patients, particularly among those in whom MAS represented the initial presentation, and was considered an important precipitating factor for MAS development [64]. When comparing patients with both SLE and MAS versus matched controls with SLE alone, MAS was associated with higher aspartate aminotransferase (AST) and lactate dehydrogenase (LDH) levels and a significant decrease in albumin. This was especially evident when MAS developed early (within three months) after SLE diagnosis, which occurred in 60% of cases [65].

Overall, MAS associated with SLE, whether occurring before or after SLE diagnosis, has been linked to increased mortality risk [63], which also correlates with low platelet counts and hyperferritinemia [64]. In this context, a clinically useful threshold with optimized sensitivity and specificity is serum ferritin > 1010 µg/L, which, when combined with fever, improves diagnostic accuracy for SLE-associated MAS [65]. Hyperferritinemia can be explained by at least two mechanisms [66]: (i) In CD68^+^ macrophages, FER2, a transcription factor regulating the ferritin H chain, binds to a site located 4.8 kb upstream of the ferritin gene transcription start site, which also contains an NF-κB binding motif. Proinflammatory cytokines such as TNF-α, IL-1, IL-2, and IL-6 further enhance ferritin synthesis. (ii) Uptake of the haptoglobin-hemoglobin (HP-Hb) complex by macrophages via CD163 stimulates intracellular ferritin production.

Finally, SLE-associated MAS may occur without overt signs of lupus activity (e.g., skin rash, peripheral edema, proteinuria). Therefore, MAS should always be considered in the differential diagnosis of persistent fever, hepatosplenomegaly, and cytopenias, particularly in the presence of elevated ferritin levels [63].

At the mechanistic level, multiple cytokines act in concert to drive the pathophysiology of MAS, forming a self-amplifying inflammatory network. IL-1β, primarily produced by activated monocytes and macrophages, plays a central role by promoting leukocyte recruitment, endothelial activation, and the release of additional pro-inflammatory mediators. Although the precise contributions of IL-6 and TNF-α to MAS remain incompletely understood, both cytokines are believed to amplify the cytokine storm, sustain systemic inflammation, and contribute to tissue injury and multiorgan dysfunction [67].

As previously stated, MAS is marked by inflammatory features and may be triggered by infections, autoimmune flares, or exogenous antigenic stimuli, including inflammatory adjuvants [58].

**Figure 1:**
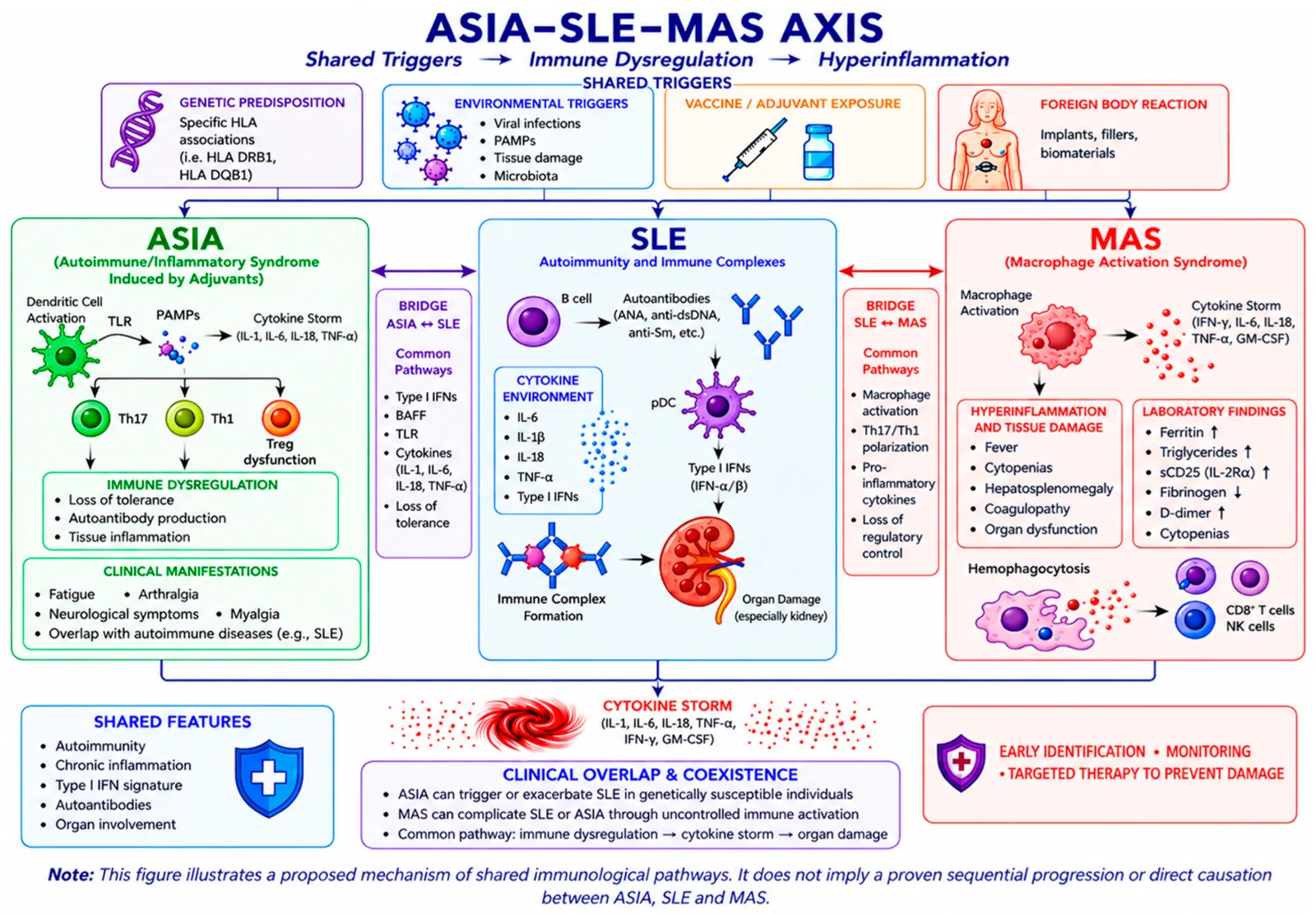
Shared and overlapping immunological pathways among SLE, ASIA, and MAS. This schematic illustrates the intersection among systemic lupus erythematosus (SLE), autoimmune/inflammatory syndrome induced by adjuvants (ASIA), and macrophage activation syndrome (MAS). Shared genetic susceptibility (e.g., HLA polymorphisms), environmental exposures, infections, vaccines/adjuvants, and foreign-body reactions may initiate innate immune activation, leading to loss of immune tolerance and chronic inflammation. In SLE, autoantibody production, immune complex deposition, plasmacytoid dendritic cell activation, and type I interferon signaling drive systemic autoimmunity and organ injury. In ASIA, activation of pattern recognition receptors by pathogen-associated molecular patterns (PAMPs) promotes dendritic cell activation, Th1/Th17 polarization, regulatory T-cell dysfunction, and pro-inflammatory cytokine production. A direct pathogenic bridge between SLE and MAS is highlighted, demonstrating that persistent type I interferon signaling, macrophage activation, CD8^+^ T-cell and natural killer (NK) cell dysfunction, amplification of the IL-18–IFN-γ axis, and excessive production of IL-1β, IL-6, TNF-α, CXCL9, and CXCL10 culminate in uncontrolled immune activation. This immune escalation may progress to a cytokine storm characterized by hemophagocytosis, hyperferritinemia, cytopenias, coagulopathy, multiorgan dysfunction, and the clinical manifestations of MAS. Together, these disorders represent an overlap of shared and interacting immunological pathways in which early recognition, biomarker monitoring, and targeted immunomodulatory therapies may prevent irreversible organ damage.

Thus, the intersection among ASIA, SLE, and MAS may be better understood as an overlap of shared and interacting immunological pathways rather than as a continuous pathogenic cascade. These conditions share mechanisms involving innate and adaptive immune activation, loss or dysregulation of immune tolerance, macrophage activation, and cytokine dysregulation. Adjuvant exposure may contribute to immune activation in susceptible individuals, whereas SLE represents a systemic autoimmune disease in which several of these pathways are also operative, and MAS represents a hyperinflammatory syndrome that may complicate SLE and other inflammatory, infectious, or neoplastic conditions. Therefore, the proposed relationship among ASIA, SLE, and MAS should be regarded as a biologically plausible conceptual framework based on overlapping immune mechanisms, without implying an established causal or sequential pathway, as demonstrated in Figure 1.

This exaggerated activation results in massive release of proinflammatory cytokines, notably IFN-γ, IL-18, TNF-α, IL-1, and IL-6, creating an intense inflammatory, autoimmune, and multiorgan-damaging environment. Consequently, multisystem involvement is observed, predominantly affecting the hematopoietic, hepatosplenic, lymphatic, and central nervous systems [58].

IL-18 levels are markedly elevated in MAS compared with other inflammatory mediators, in contrast to rheumatologic diseases in which elevations are generally moderate. IL-18 is a potent stimulator of NK cells to produce IFN-γ; severe imbalance of IL-18 may lead to uncontrolled activation of macrophages and T lymphocytes that escape NK-cell–mediated immunoregulation [67].

Moreover, IFN-γ stimulates the production of proinflammatory cytokines such as IL-6, IL-12, and IL-23. Evidence indicates that sustained IFN-γ stimulation is a key driver of macrophage activation and hemophagocytosis observed in MAS [67].

Additionally, Moling et al. reported the bridging of MAS, ASIA, and intravascular large B-cell lymphoma, established through clinical/laboratory criteria and histologic analysis [68]. In that case, criteria for MAS were met without an evident etiology; thus, MAS could have resulted from the lymphoma or, alternatively, from ASIA related to a silicone breast implant, despite the absence of prior scientific confirmation of this association. The authors therefore proposed hypothetical interactions among these syndromes, illustrated below [68].

As suggested, ASIA, initiated by a foreign-body reaction to silicone and persistent inflammatory stimulation, may trigger MAS directly or indirectly in a genetically permissive setting. Importantly, MAS detection is inherently difficult and can be further complicated by overlapping manifestations shared with ASIA, such as splenomegaly and pyrexia [68]. Other triggers may also precipitate MAS, including infections (viral, bacterial, parasitic, and fungal), autoimmune diseases (in addition to SLE, the most common are adult-onset Still’s disease, systemic juvenile idiopathic arthritis, and Kawasaki disease), malignancies (both hematologic and solid tumors, particularly Hodgkin lymphoma, leukemia, and multicentric Castleman disease), cell-based therapies, and certain medications [66].

Based on the discussion above, the three conditions, SLE, MAS, and ASIA, can be understood as a mutually reinforcing cycle, sharing mechanisms and inflammatory mediators that promote sustained inflammation and autoimmunity, as shown in Figure 1.

Therefore, in MAS, dysregulated activation of macrophages, dendritic cells, and T lymphocytes leads to increased IL-18, IL-1β, and TNF-α, which in turn promote IL-6, IL-23, and IL-12 production, sustaining inflammation through a “cytokine storm” [66,67]. IL-18, IL-12, and IFN-γ also contribute to Th1 differentiation, a helper T-cell subset that activates macrophages. In addition, elevated IL-6, IL-23, and IL-1β stimulate IL-17 production by Th17 cells, reinforcing inflammation, antibody production, and macrophage recruitment [66,67]. Collectively, these pathways not only perpetuate MAS itself but also enhance antigen presentation (by activated macrophages and dendritic cells) and autoantibody production, thereby maintaining autoimmunity, already facilitated in ASIA, particularly in the presence of a favorable genetic background such as SLE.

With respect to ASIA, in response to the adjuvant-driven activation of innate immunity (recruitment of macrophages, monocytes, dendritic cells, and neutrophils) and adaptive immunity (Treg dysfunction and antibody production), cytokines such as IL-4, IL-5, and IL-13 are produced, promoting B-cell differentiation into plasma cells and, consequently, autoantibody generation [9,14]. Notably, experimental models suggest that IL-4, part of the ASIA cytokine loop, may favor the anti-DNA/chromatin axis and thereby facilitate autoimmune disease development [35,36,37,38,39]. This helps explain how ASIA may trigger SLE, as supported by the prior literature, and also highlights the overlap between ASIA-related immune hyperactivation and the dysregulated immune activation characteristic of MAS.

Even mediators more closely linked to SLE contribute to this reinforcing loop. Increased IL-10 and IL-21 promote autoimmunity, whereas reduced IL-2 impairs Treg function, thereby exacerbating both ASIA-associated hyperresponsiveness and MAS-associated macrophage hyperactivation [5,6,8]. IL-10, like TGF-β, may play a more nuanced role: despite supporting autoantibody production, it can inhibit certain inflammatory pathways [69]. However, because IL-10 is produced by macrophages and dendritic cells (dysregulated in MAS) and by Tregs (impaired due to IL-2 deficiency and ASIA-related mechanisms), its counter-regulatory role may be insufficient to halt the pathogenic process.

Overall, IL-6 and IL-23 appear to be key shared contributors across these syndromes, as both drive Th17 differentiation and sustained IL-17 production, thereby maintaining a proinflammatory milieu [70]. Moreover, IL-6 promotes B-cell proliferation and leukocyte recruitment, whose downstream effects contribute to tissue injury in concert with IL-1, TNF-α, IL-17, and IFN-γ [71]. Notably, B-cell-derived IL-6 has been shown to promote the formation of spontaneous autoimmune germinal centers in a murine model of SLE, thereby contributing to disease pathogenesis. These autoimmune germinal centers represent an important source of autoantibody-producing plasma cells in SLE and potentially in other humoral autoimmune diseases. Mechanistically, IL-6 produced by B cells promotes the transient expression of B-cell lymphoma 6 protein (BCL-6), the master transcriptional regulator of T follicular helper, in cognate CD4^+^ T cells [72]. This signaling promotes T follicular helper differentiation and, consequently, spontaneous germinal center formation. The process is further enhanced by IFN-γ, which is likely produced locally by activated T helper 1 (Th1) cells.

**Figure 2:**
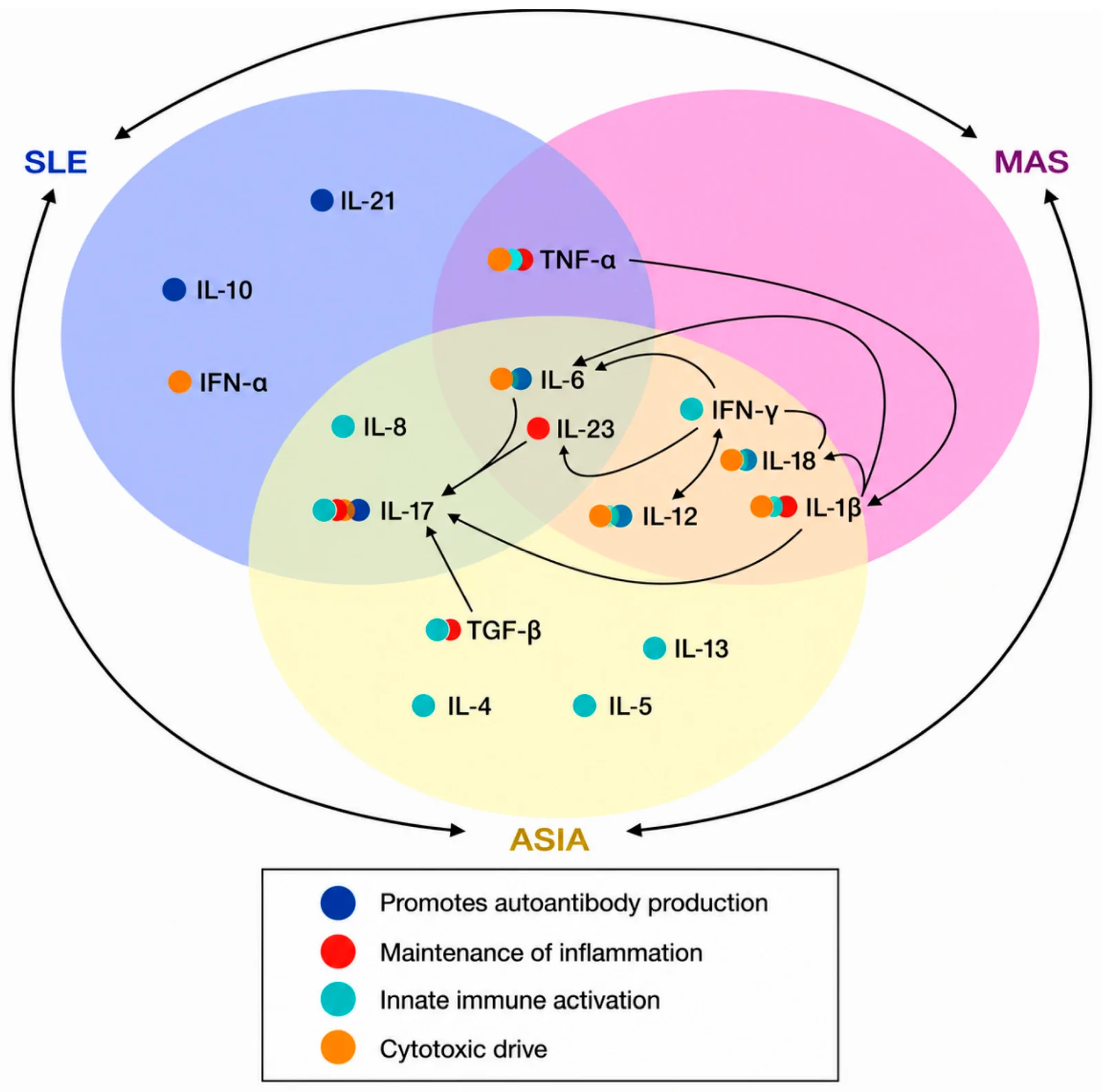
Cytokine network linking systemic lupus erythematosus (SLE), autoimmune/inflammatory syndrome induced by adjuvants (ASIA), and macrophage activation syndrome (MAS). The Venn diagram illustrates the distribution of major cytokines across SLE, ASIA, and MAS, highlighting shared and disease-specific inflammatory pathways. Cytokines located within overlapping regions represent mediators implicated in more than one condition, whereas those positioned within individual circles are more closely associated with disease-specific immune responses. Curved arrows indicate functional interactions and amplification loops among cytokines that contribute to immune dysregulation and progression toward hyperinflammation. Colored dots adjacent to each cytokine denote their principal immunological functions: promotion of autoantibody production (blue), maintenance of inflammation (red), innate immune activation (cyan), and cytotoxic drive (orange). Collectively, the figure emphasizes the convergence of innate and adaptive immune pathways centered on IL-1β, IL-6, TNF-α, IFN-γ, IL-18, IL-12, and IL-23, supporting the concept of a shared cytokine network that mechanistically links SLE, ASIA, and MAS.

Figure 2 summarizes the shared pathophysiology among the three conditions.

## 4. Limitations

This review has several limitations. First, given its narrative design, the selection and interpretation of the available literature were not based on a systematic review methodology, which may introduce selection bias. Second, the clinical evidence supporting a direct association between ASIA and SLE remains limited and is largely derived from observational studies, case series, and case reports, while epidemiological findings are heterogeneous. Therefore, the proposed relationship among ASIA, SLE, and MAS should be interpreted as a conceptual framework based on shared and overlapping immunological mechanisms rather than as an established causal or sequential pathway. Further prospective and mechanistic studies are needed to determine whether these immunological associations translate into clinically relevant causal relationships.

## 5. Conclusions

The evidence reviewed supports the concept that ASIA, SLE, and MAS share overlapping clinical and immunological features and convergent immune pathways. These conditions may involve common mechanisms of immune dysregulation, particularly in genetically susceptible individuals exposed to environmental, infectious, or adjuvant-related stimuli. However, the available evidence does not establish a causal or sequential relationship among ASIA, SLE, and MAS. Rather, clinical and experimental findings support mechanistic overlap based on shared mechanisms, including activation of innate and adaptive immunity, loss of immune tolerance, macrophage activation, and dysregulation of cytokine networks involving type I interferons, IL-1β, IL-6, IL-18, TNF-α, and IFN-γ.

Recognition of the intersection among ASIA, SLE, and MAS provides an integrated framework for understanding their shared immunological pathways and overlapping inflammatory phenotypes. This perspective highlights the importance of carefully assessing previous exposure to adjuvants or biomaterials, integrating clinical manifestations with laboratory biomarkers, and maintaining a high index of suspicion for MAS in patients with active SLE who develop persistent fever, hyperferritinemia, cytopenias, or multiorgan dysfunction. Early diagnosis and timely immunomodulatory treatment remain critical to reducing morbidity and mortality.

Future studies should focus on validating biomarkers capable of identifying individuals susceptible to immune dysregulation and severe inflammatory phenotypes, further characterizing the shared and disease-specific molecular mechanisms underlying ASIA, SLE, and MAS, and evaluating targeted therapeutic strategies directed against common inflammatory pathways. A deeper understanding of these overlapping mechanisms and their clinical relevance may improve risk stratification, refine the characterization of immune-mediated phenotypes, and support the development of precision medicine approaches for patients with autoimmune and hyperinflammatory disorders.

An important translational gap remains between experimental evidence and the establishment of causality in humans. Future mechanistic studies should therefore incorporate in vitro models using human immune cells exposed to conventional and next-generation adjuvants to determine whether the immune alterations associated with ASIA, SLE, and MAS can be reproduced under controlled conditions. Such approaches could characterize shared and disease-specific features of innate immune activation, loss of immune tolerance, macrophage activation, T- and B-cell responses, and cytokine dysregulation, thereby helping to define the extent of mechanistic overlap among these conditions without presupposing a causal or sequential relationship. These studies could be complemented by spatial biology approaches, including spatial transcriptomics and highly multiplexed spatial proteomics, to characterize the tissue-level organization of immune responses while preserving anatomical context and cell–cell interactions. Spatial profiling of affected tissues could help identify the localization, activation states, and interactions of macrophages, dendritic cells, T and B lymphocytes, and tissue-resident cells, while simultaneously mapping shared and disease-specific inflammatory and immune-regulatory pathways. Integration of spatial omics with single-cell technologies could further resolve cellular heterogeneity and identify disease-associated cellular neighborhoods and signaling networks that may not be detectable through conventional bulk analyses. In parallel, prospective studies integrating soluble biomarkers, including cytokine and chemokine profiles, with emerging molecular diagnostics such as transcriptomic signatures and targeted gene-expression panels may facilitate the identification of immune dysregulation and improve risk stratification. Ultimately, the integration of in vitro human models, spatial and single-cell biology, and circulating biomarkers may provide a multidimensional framework for evaluating the biological rationale and clinical relevance of the immunological overlap among ASIA, SLE, and MAS and for identifying individuals susceptible to severe inflammatory phenotypes. These candidate biomarkers and molecular signatures, however, require rigorous prospective validation before their diagnostic or prognostic utility can be established.

Additionally, the development of an integrated investigative framework may represent an important future direction for investigating patients with overlapping clinical and immunological features of ASIA, SLE, and MAS. Currently, these conditions are assessed independently using distinct frameworks, including the proposed major and minor criteria for ASIA, the 2019 EULAR/ACR classification criteria for SLE, and tools such as the HScore and HLH-2004 criteria for MAS/secondary HLH. A future framework could incorporate complementary domains reflecting adjuvant exposure and host susceptibility, autoimmune manifestations and serological markers, and hyperinflammatory features such as fever, cytopenias, hyperferritinemia, hypertriglyceridemia, hypofibrinogenemia, organ dysfunction, and markers of macrophage activation. Emerging immunological and molecular biomarkers could eventually complement these clinical domains. However, given the limited clinical evidence supporting direct relationships among ASIA, SLE, and MAS and the absence of prospective validation, such a framework should not be interpreted as evidence that these conditions constitute a unified disease spectrum or follow a causal or sequential pathway. Accordingly, assigning numerical weights or diagnostic cutoffs would be premature. Future multicenter prospective studies should determine whether an integrated approach to patients with overlapping features provides incremental diagnostic or prognostic value beyond existing disease-specific criteria and should establish its sensitivity, specificity, calibration, and external validity before any clinical implementation.

In conclusion, this review proposes a conceptual framework based on the shared and overlapping immunological pathways among ASIA, SLE, and MAS. Rather than implying an established causal or sequential relationship, this framework highlights areas of biological basis and mechanistic convergence, including innate and adaptive immune activation, loss of immune tolerance, macrophage activation, and cytokine dysregulation. This perspective may provide a basis for future translational and clinical studies aimed at determining the clinical relevance of these immunological overlaps and improving the characterization, monitoring, and management of patients with complex autoimmune and hyperinflammatory disorders.

## Figures and Tables

**Table 1:** Bioimplant-induced ASIA and SLE.

Study	Year	Patient Demographics	Adjuvant Exposure	Clinical Manifestations	Diagnostic Criteria Met	Treatment	Outcome
Knapik et al. [50]	2024	26-year-old woman	Tattoo ink, hyaluronic acid, piercing	Arthralgia, fever, butterfly rash, erythematous skin lesions on thighs/buttocks, delayed inflammatory reaction to hyaluronic acid	ACR/EULAR 2019 SLE criteria; ASIA criteria	Antibiotics, NSAIDs, steroids, hydroxychloroquine, cyclosporine	Improvement in general condition, resolution of swelling and joint pain, improvement in skin lesions
Mir et al. [51]	2022	37-year-old woman	Silicone breast implants	Mucocutaneous, musculoskeletal, hematological, neurological, and renal involvement	ASIA criteria; SLE diagnosis	Not specified	Not specified
Vitiello et al. [52]	2026	42-year-old man	Oil mineral injection	Hematological, neurological, and kidney involvement	ASIA criteria; SLE and lupic nephritis diagnosis; MAS	Cyclophosphamide, steroids, antibiotics, hemodialysis	Clinical and laboratory recovery

ACR = American College of Rheumatology; EULAR = European Alliance of Associations for Rheumatology; ASIA = Autoimmune/Inflammatory Syndrome Induced by Adjuvants; SLE = Systemic Lupus Erythematosus; MAS = Macrophage Activation Syndrome; NSAIDs = Nonsteroidal Anti-Inflammatory Drugs.

**Table 2:** Vaccine-induced ASIA and SLE (Large-Scale Studies and Individual Case Report).

Study	Year	Study Design	Sample Size	Vaccines Studied	Main Findings	Risk Estimate
Xie et al. [53]	2026	Pharmacovigilance analysis (VAERS)	11,301,263 AEs; 1976 vaccine-associated lupus cases	HPV, HBV, COVID-19, influenza, zoster	Vaccine-associated lupus uncommon (0.017%); median onset 8 days; HPV and HBV vaccines associated with lupus AEs; COVID-19, influenza, zoster showed no relationship	HPV and HBV: significant association; COVID-19, influenza, zoster: no association
Wang et al. [54]	2024	Meta-analysis	17 studies; 45,067,349 individuals	HBV, HPV, influenza, COVID-19	Overall no significant association; HBV significantly associated; HPV slightly associated; influenza no association; COVID-19 marginally protective	Overall OR 1.14 (0.86–1.52); HBV OR 2.11 (1.11–4.00); HPV OR 1.43 (0.88–2.31)
Wang et al. [55]	2017	Meta-analysis	12 studies on SLE	Various vaccines	Vaccinations significantly increased risk of SLE; stronger association with short vaccination time	Overall RR 1.50 (1.05–2.12); short vaccination time RR 1.93 (1.07–3.48)
Grimaldi-Bensouda et al. [56]	2014	International case–control study	105 SLE patients; 712 controls	Various vaccines	No association between vaccination and SLE risk	Adjusted OR 0.9 (0.5–1.5) for 24 months; similar for 12 months
Arévalo-Cañas et al. [57]	2025	85-year-old woman	Shingrix (recombinant zoster vaccine)	Not Specified	Pleuropericarditis with pleural and pericardial effusion.	ANA 1:640 (homogeneous), IgG anti-cardiolipin positive, symptom resolution within 2 months; ANA and anti-cardiolipin negative at 1 year

AEs = Adverse Events; HBV = Hepatitis B Virus; HPV = Human Papillomavirus; COVID-19 = Coronavirus Disease 2019; OR = Odds Ratio; RR = Relative Risk; ANA = Antinuclear Antibodies.

## Data Availability

No new data were generated or analyzed in this study. Data sharing is not applicable to this article, as all information presented was obtained from previously published studies and publicly available sources cited in the manuscript.

## Abbreviations

ANAs, antinuclear antibodies; anti-dsDNAs, anti-double-stranded DNA antibodies; anti-Sms, anti-Smith antibodies; ASIA, autoimmune/inflammatory syndrome induced by adjuvants; BAFF, B-cell activating factor; CD8, cluster of differentiation 8-positive T lymphocytes; CXCL9, C-X-C motif chemokine ligand 9; CXCL10, C-X-C motif chemokine ligand 10; DAMPs, damage-associated molecular patterns; GM-CSF, granulocyte–macrophage colony-stimulating factor; HLA, human leukocyte antigen; IFN, interferon; IFN-α, interferon alpha; IFN-β, interferon beta; IFN-γ, interferon gamma; IL, interleukin; IL-1β, interleukin-1 beta; IL-6, interleukin-6; IL-18, interleukin-18; MAS, macrophage activation syndrome; MCP-1, monocyte chemoattractant protein-1 (CCL2); NK cells, natural killer cells; PAMPs, pathogen-associated molecular patterns; pDC, plasmacytoid dendritic cell; sCD25, soluble interleukin-2 receptor alpha (soluble CD25); SLE, systemic lupus erythematosus; Th1, T helper 1 cell; Th17, T helper 17 cell; TLR, Toll-like receptor; TNF-α, tumor necrosis factor-alpha; Treg, regulatory T cell.
